# Alzheimer's Disease-Linked Mutations in *Presenilin-1* Result in a Drastic Loss of Activity in Purified γ-Secretase Complexes

**DOI:** 10.1371/journal.pone.0035133

**Published:** 2012-04-18

**Authors:** Matthias Cacquevel, Lorène Aeschbach, Jemila Houacine, Patrick C. Fraering

**Affiliations:** École Polytechnique Fédérale de Lausanne, Brain Mind Institute, Laboratory of Molecular and Cellular Biology of Alzheimer's Disease, Lausanne, Switzerland; University G. D'Annunzio, Italy

## Abstract

**Background:**

Mutations linked to early onset, familial forms of Alzheimer's disease (FAD) are found most frequently in *PSEN1*, the gene encoding presenilin-1 (PS1). Together with nicastrin (NCT), anterior pharynx-defective protein 1 (APH1), and presenilin enhancer 2 (PEN2), the catalytic subunit PS1 constitutes the core of the γ-secretase complex and contributes to the proteolysis of the amyloid precursor protein (APP) into amyloid-beta (Aβ) peptides. Although there is a growing consensus that FAD-linked PS1 mutations affect Aβ production by enhancing the Aβ1–42/Aβ1–40 ratio, it remains unclear whether and how they affect the generation of APP intracellular domain (AICD). Moreover, controversy exists as to how PS1 mutations exert their effects in different experimental systems, by either increasing Aβ1–42 production, decreasing Aβ1–40 production, or both. Because it could be explained by the heterogeneity in the composition of γ-secretase, we purified to homogeneity complexes made of human NCT, APH1aL, PEN2, and the pathogenic PS1 mutants L166P, ΔE9, or P436Q.

**Methodology/Principal Findings:**

We took advantage of a mouse embryonic fibroblast cell line lacking PS1 and PS2 to generate different stable cell lines overexpressing human γ-secretase complexes with different FAD-linked PS1 mutations. A multi-step affinity purification procedure was used to isolate semi-purified or highly purified γ-secretase complexes. The functional characterization of these complexes revealed that all PS1 FAD-linked mutations caused a loss of γ-secretase activity phenotype, in terms of Aβ1–40, Aβ1–42 and APP intracellular domain productions *in vitro*.

**Conclusion/Significance:**

Our data support the view that PS1 mutations lead to a strong γ-secretase loss-of-function phenotype and an increased Aβ1–42/Aβ1–40 ratio, two mechanisms that are potentially involved in the pathogenesis of Alzheimer's disease.

## Introduction

Since their discovery in 1995 and their association with early onset familial Alzheimer's disease (FAD) [1], [2], the presenilin genes *PSEN1* and *PSEN2* have been widely studied, and the complexity of their biological role is becoming increasingly evident. *PSEN1* and *PSEN2* encode transmembrane proteins PS1 and PS2, respectively, that constitute the catalytic core of γ-secretase, the founding member of an emerging class of unconventional, Intramembrane-Cleaving Proteases (I-CLiPs) [3]. Active γ-secretase is a multiprotein complex composed of PS1 or PS2 together with nicastrin (NCT), the anterior pharynx-defective protein 1 (APH1), and the presenilin enhancer 2 (PEN2). Experimental evidence such as the binding of transition-state analogue γ-secretase inhibitors to PS1 [4], as well as the abolishment of γ-secretase activity when PS1 lacks the aspartate residues critical for proteolysis [4], [5], [6], have confirmed that presenilins harbour the active site of the enzymatic complex.

PS1 and PS2 play fundamental roles in cell signalling as part of the γ-secretase complex. The latter cleaves numerous type-I membrane proteins in their transmembrane domain [7], releasing their corresponding intracellular domains, which are capable of influencing gene expression. For some substrates like NOTCH1 or ERBB4, the γ-secretase cleavage is essential for their biological function, whereas for other substrates like DCC or NEUREXIN-3β [8], the possible role of the cleavage in signalling events has not been formally established (see [9] for a review). The amyloid precursor protein (APP) is processed by the successive actions of β-secretase (BACE1) and γ-secretase, generating amyloid-beta peptides (Aβ) of different lengths, ranging from 37 to 46 amino acids [10]. Cleavage of the APP C-terminal fragments (APP-CTFs) by γ-secretase also releases the APP intracellular domain (AICD), which has been recently involved in the regulation of brain ApoE expression, a major genetic determinant of AD, and in cholesterol metabolism [11]. In addition, PS1 has been shown to interact with a growing list of proteins that modulate γ-secretase activity [9], [12], [13], [14].

In a pathological context, 185 missense mutations in *PSEN1* and 13 mutations in *PSEN2* have been identified and found to be associated with FAD (www.molgen.ua.ac.be/ADMutations). It was initially suggested that such mutations lead to a toxic gain of function because they are associated with a relative increased production of longer and more hydrophobic Aβ species, mainly Aβ1–42, that accumulate and aggregate early in the course of the disease [15], [16]. Similar phenotypes have been observed by independent groups in cell-based studies performed with different mammalian cell lines [17], [18], [19], [20], [21], [22]. Despite this growing consensus, the above-mentioned studies provided conflicting results regarding the relative proportions of Aβ42, Aβ40, and AICD resulting from the *PSEN* mutations. On one hand, a gain-of-function phenotype was suggested due to the observed increase in Aβ42 production, accompanied or not with reduced Aβ40 production, thereby leading to an increased Aβ42/Aβ40 ratio. On the other hand, reduced AICD production suggested a loss-of-function phenotype [20]. Differences in the methodologies and cell types used to assess the effects of *PSEN* mutations on Aβ and AICD productions can explain this controversy as to how PS mutations exert their effects. For example, endogenous PS has been suggested to affect such analysis, as the FAD-linked *PSEN1* mutations led to a decrease in the total amount of Aβ generated in PS1 and PS2 double knockout cells [23]. Next, it has been reported that γ-secretase complexes are heterogeneous in composition (with two PS genes and three APH1 isoforms, six combinations of γ-secretase are possible), with distinct functional properties influencing the relative amount of Aβ species generated [24]. In order to investigate the biochemical and functional properties of γ-secretase in cell-free systems, we and others have recently purified this enzymatic complex to homogeneity [25], [26], [27]. In particular, protocols for the high-grade purification of proteolytically active γ-secretase constituted of NCT, APH1aL, PEN2 and wild-type PS1 [26], [27] allowed the reconstitution of 3D structures at 15 Å and 12 Å resolution by EM and cryo-EM and gave new insights on the structure and activity of the enzyme [28], [29]. However, these studies were exclusively focused on the wild-type PS1. Here, we report for the first time the functional characterization of highly purified and homogenous human γ-secretase particles carrying different FAD-linked PS1 mutants. Our biochemical and functional findings strongly suggest that pathogenic mutations in *PSEN1* cause a loss of γ-secretase activity.

## Results

### Generation, selection and characterization of stable cell lines overexpressing γ-secretase complexes with FAD-linked PS1 mutants

In order to facilitate high-grade purification of homogenous human γ-secretase complexes containing different PS1 variants, we took advantage of a previously generated mouse embryonic fibroblast (MEF) cell line that lacks the two presenilins (PS1/2^−/−^) [30], [31]. Our global strategy consisted in producing stable cell lines, on a PS1/2^−/−^ background, that overexpress tagged versions of the three human γ-secretase subunits NCT, APH1 and PEN2 together with different variants of human PS1, and to purify the different human γ-secretase complexes by three sequential affinity purification steps. This strategy allowed us to exclude a possible co-purification of mouse γ-secretase components. First, a parental cell line was generated by co-transducing lentiviral vectors of human NCT-V5 (hNCT-V5), human APH1aL-HA (hAPH1aL-HA) and human Flag-PEN2 (Flag-hPEN2) into MEF PS1/2^−/−^. Lentivectors are stably integrated into the cell genome and allow the generation of cell lines overexpressing multiple genes in a short period of time. Next, the clone expressing the highest levels of all three recombinant proteins was selected (designated γ - PS1/2) and used as the parental cell line that was further transduced with lentivectors carrying the following different human PS1 variants: PS1-WT, three dominant-negative forms of PS1 that lack the crucial, catalytic site aspartate residues (D257A, D385A, and both D257A/D385A referred to as DDAA later in the article), and three FAD-linked PS1 mutants (L166P, ΔE9 and P436Q, with mean disease onset at 24 years, 45.5 years and 28.3 years, respectively).

**Figure 1:**
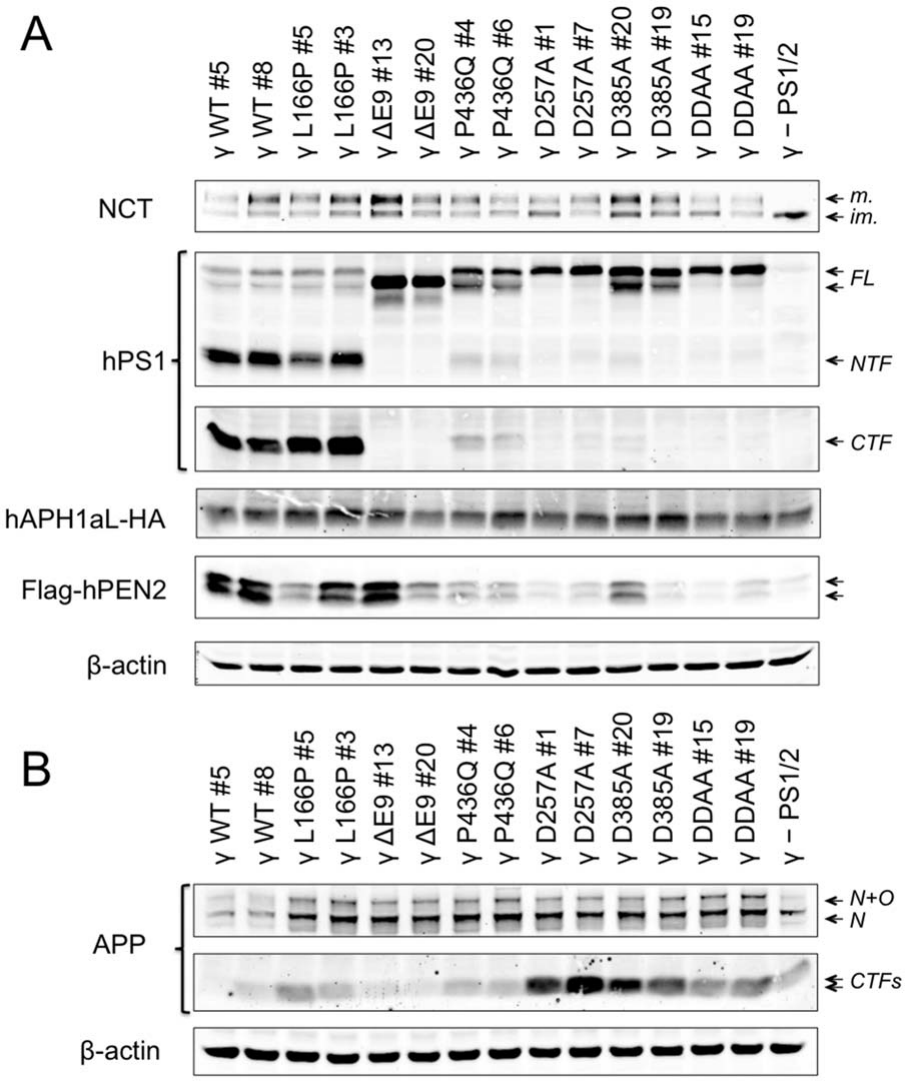
Generation of stable cell lines overexpressing all human γ-secretase components with FAD-linked PS1 variants. MEF PS1/2^−/−^ were stably co-transduced with lentiviral vectors carrying genes encoding hNCT-V5, Flag-hPEN2, hAPH1aL-HA and clones were isolated by limiting dilution to generate a cell line, designated as γ- PS1/2, that overexpresses high amount of the three subunits. γ- PS1/2 MEFs were further transduced with hPS1 variants harbouring FAD-linked mutations or mutations in the catalytic aspartate residue(s), or PS1-WT, and cloned. Each clone, derived form the γ- PS1/2, was conveniently named according to the mutation present in PS1 preceded by the symbol γ and followed by the number of the clone (γ-MEF) in order to distinguish them from wild-type MEF (WT MEF) and MEF PS1/2^−/−^. Two clones per γ-secretase variant were selected for characterization. (A–B) Whole cell protein extracts of the different cell lines were prepared in 1% NP40-HEPES buffer, separated by SDS-PAGE on 4–12% Bis-Tris or 12% Tris-Glycine gels and analysed by immunostaining to detect the γ-secretase core components NCT (NCT164), PS1 (NTF, MAB1563; CTF; MAB5232), APH1aL-HA (3F10), and Flag-PEN2 (M2) (A), and endogenous APP (A8717) (B). β-Actin was used as a loading control. Each lane represents one selected clone. *CTF*: C-terminal fragment, *FL*: full-length, *im.*: immature NCT; *m.*: mature NCT, *N*: N-glycosylated, *NTF*: N-terminal fragment, *O*: O-glycosylated.

For each PS1 variant, two individual clones were selected based on the expression levels of the recombinant proteins, and extensively characterized (Figure 1). To distinguish those cell lines overexpressing all γ-sectease components from wild-type MEFs, they are collectively designated γ-MEFs. As shown in Figure 1A, the analysis of total protein extracts confirmed that PS is required for the maturation of NCT and the stability of PEN2 [23], [32]. As expected, and when compared to the parental γ- PS1/2 cell line, higher levels of the mature form of NCT were observed in all PS1-overexpressing clones. In contrast to PEN2, APH1aL levels were relatively similar in all PS1 clones and the parental cell line. Indeed, and as previously reported [23], [32], PEN2 levels were higher in the clones overexpressing WT PS1 and FAD-linked PS1 mutants, and to a lesser extent in the clones overexpressing the dominant negative forms of PS1. Interestingly, PEN2 migrated on the gel as an apparent double band, possibly reflecting a post-translational modification. Different patterns of PS1 expression and autoproteolysis were observed in the different clones. First, and in sharp contrast to the other clones, those expressing PS1-WT or PS1-L166P displayed higher levels of PS1-NTF and PS1-CTF fragments compared with full-length PS1 (PS1-FL; Figure 1A). As estimated by densitometry, the NTF fragments of PS1-WT and PS1-L166P account for 87±2% and 79±7% of total PS1 (PS1-NTF+PS1-FL), respectively. Next, the PS1-ΔE9 clones did not show any detectable NTF or CTF fragments, as previously described [33], while P436Q and the three dominant negative variants of PS1 were characterized by low levels of PS1-NTF and PS1-CTF fragments, suggesting that these variants were less prone to endoproteolysis.

We next investigated the effects of *PSEN1* mutations on the processing of endogenous APP. As expected, and as indicated by increased levels of intracellular APP-CTFs (a common characteristic among known substrates in response to γ-secretase pharmacological inhibition), the activity of γ-secretase was significantly altered in the PS deficient parental γ - PS1/2 cell line as well as in all clones expressing dominant negative forms of PS1 (Figure 1B). In the clones expressing PS1-WT, APP-CTFs were almost undetectable due to high γ-secretase activity, while clones expressing PS1-L166P or PS1-P436Q displayed APP-CTF accumulation. In PS1-ΔE9 clones, a mild APP-CTF accumulation was observed (Figure 1B). Although a poor recovery of γ-secretase activity for the PS1-L166P and a comparatively better performance of PS1-ΔE9 have been reported by others under similar experimental conditions [23], the potential of PS1-P436Q mutation to restore the processing of endogenous APP-CTFs has never been assessed [34], [35]. Importantly, all observations described above for the cells overexpressing simultaneously hNCT-V5, hAPH1aL-HA, Flag-hPEN2 and the different PS1 variants are very consistent with those made in MEF PS1/2^−/−^ cells overexpressing only the PS1 variants, in the absence of the other γ-secretase components (cf. Figure S1A). These data suggest that the effect of FAD mutations in PS1 on APP-CTFs accumulation was similar in presence or absence of other human components of the γ-secretase.

**Figure 2:**
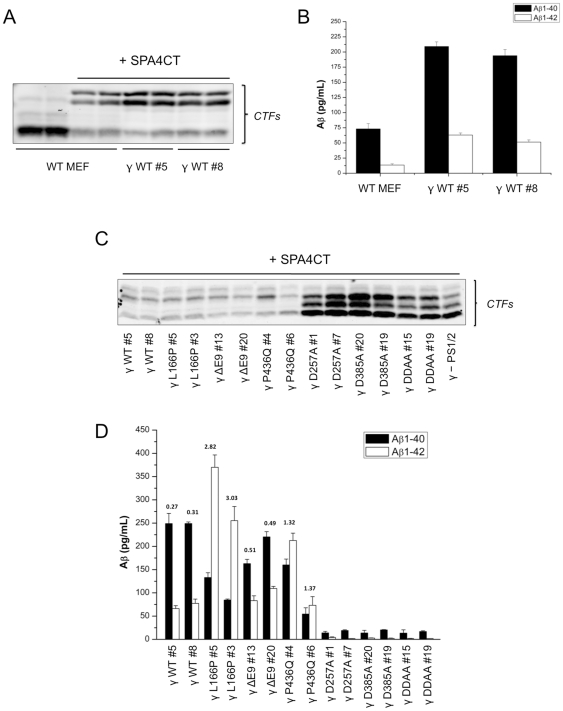
Aβ production in cell lines overexpressing human γ-secretase components with FAD-linked PS1 variants. WT MEF, γ-MEF and γ - PS1/2 were transduced with an APP-based substrate corresponding to the 99 C-terminal residues of human APP fused to the APP signal peptide in N-terminus (SPA4CT [36]) and a Flag Tag in C-terminus. Cell proteins were extracted in 1% NP40-HEPES buffer, separated by SDS-PAGE on 12% Tris-Glycine gels and analysed by immunostaining with an antibody targeting the C-terminal part of APP (A8717) (A, C). Aβ1–40 and Aβ1–42 levels were also measured in the corresponding cell culture media (B, D). Data corresponds to three independent experiments (Mean ± SEM).

Next, we further investigated the effect of PS1 variants on the cellular production/secretion of Aβ species. For this purpose, and as described elsewhere [36], we used an APP-based γ-secretase substrate with a Flag tag at its C-terminus (SPA4CT-Flag). This substrate was transduced in the above-described γ-secretase overexpressing stable cell lines (γ-MEFs) as well as in wild-type MEFs, and APP-CTFs and Aβ levels were measured in cell lysate and cell media, respectively (Figure 2). Under these conditions, the overexpression of SPA4CT-Flag led to an accumulation of two bands corresponding to the exogenous substrate, associated with a concomitant decrease of endogenous APP-CTFs levels (Figure 2A and Figure S2). As estimated by ELISA, a three-fold increase in Aβ1–40 and Aβ1–42 levels was observed in the cell culture media of cell lines overexpressing human γ-secretase (γ-MEF WT), in comparison to untransduced WT MEFs (Figure 2B). Also, the average Aβ1–42/Aβ1–40 ratios were not significantly different in those cell lines (Mean ± SD: γ-MEF WT#5: 0.30±0.01; γ-MEF WT#8: 0.29±0.01; WT MEF: 0.19±0.06). Next, overexpression of SPA4CT-Flag in γ-MEFs led to the pronounced intracellular accumulation of APP-CTFs in cells overexpressing the PS1 aspartate mutants (Figure 2C), and to the secretion in the cell culture media of different Aβ levels as measured by ELISA (Figure 2D). First, only traces of both Aβ1–40 and Aβ1–42 were detected in γ-MEFs overexpressing PS1 with aspartate mutants. Next, γ-MEFs overexpressing PS1 with FAD-linked mutations displayed a pronounced variability in Aβ levels when compared to the wild-type clones. Overall, the average Aβ1–40 levels were decreased in these cell lines (Mean of two clones in pg/mL ± SD: WT: 224.6±0.1, L166P: 84.3±34.2, ΔE9: 167.2±40.7, P436Q: 82.8±74.7), while the Aβ1–42 levels were increased (Mean of two clones in pg/mL ± SD: WT: 69.9±7.9, L166P: 310.7±81.0, ΔE9: 94.4±18.5, P436Q: 141.1±98.5). Consistent with previously reported data, the Aβ1–42/Aβ1–40 ratios were increased in all γ-MEF clones overexpressing PS1 with FAD-linked mutations (Figure 2D) (Mean of two clones pg/mL ± SD: WT: 0.31±0.03, L166P: 3.8±0.58, ΔE9: 0.6±0.03, P436Q: 2.0±0.59).

### FAD-linked PS1 mutations alter the activity of semi-purified γ-secretase complexes

**Figure 3:**
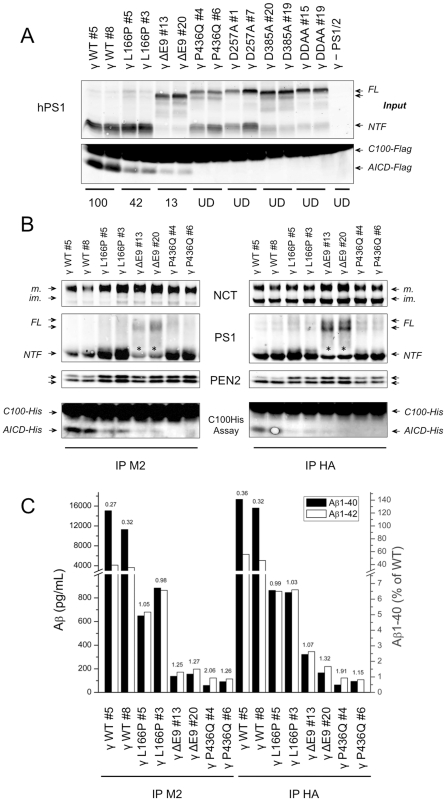
Enzymatic activity of partially purified γ-secretase complexes with FAD-linked PS1 mutants. (A) γ-Secretase activity assays performed with γ-MEF and γ - PS1/2 microsomal extracts prepared in 1% CHAPSO-HEPES buffer. Equal protein levels from the different extracts were diluted to 0.25% CHAPSO-HEPES buffer and incubated for 4 h at 37°C with lipids and 1 µM of recombinant human APP-based substrate (C100-Flag). Samples were analyzed by SDS-PAGE and immunostained with anti-Flag (M2) or anti-PS1 (MAB1563). The relative amounts of AICD-Flag generated in the reactions, reflecting γ-secretase activity, were estimated by densitometry. PS1 immunostaining was used to assess the amount of input material. (B) Equal amounts of microsomal proteins were immunoprecipitated overnight at 4°C with either anti-Flag M2 or anti-HA affinity resins, and submitted to a C100-His assay according to the same protocol as in (A). Protein samples were separated by SDS-PAGE and analysed by immunostaining for γ-secretase subunits ((NCT164 (NCT), MAB1563 (PS1-NTF), or UD1 (PEN2)). AICD-His cleavage products were immunostained with an anti-APP-CTF antibody (A8717). *Indicates a non-specific band corresponding to the IgG light chains. (C) Aβ1–40 and Aβ1–42 were quantified by sandwich ELISA and represented in pg/mL (left Y-axis) or in percentage (right Y-axis) of the mean of Aβ1–40 levels generated by the two wild-type clones. Aβ1–42/Aβ1–40 ratios are indicated on the top of the bars. The results were confirmed in three independent experiments and a representative dataset is shown.

We next assessed the activity of γ-secretase with FAD-linked PS1 mutations in microsomal extracts of γ-MEFs. Membrane protein extracts were prepared and γ-secretase activity was assayed on an exogenous recombinant APP-based substrate consisting of the C-terminal 99 amino acid residues of APP, and referred to as C100-Flag (Figure 3A). The generation of the cleavage product AICD-Flag was detected by Western blot analysis and measured by densitometry. As shown in Figure 3A, the levels of AICD-Flag generated from the PS1-L166P and PS1-ΔE9 clones accounted for 42±1% and 13±6% of that in the PS1-WT clones, respectively. AICD-Flag was undetectable in the clones expressing PS1-P436Q as well as in the three dominant negative aspartate PS1 variants. Similar results were observed in MEF PS1/2^−/−^ cells overexpressing only the PS1 variants, in the absence of the other human γ-secretase components (Figure S1B).

We next performed activity assays under semi-purified conditions. To do so, γ-secretase complexes from the different clones were co-immunoprecipitated from microsomal extracts and their activities were assayed *in vitro* with the C100-His substrate. Two immunoprecipitation protocols using two affinity resins (anti-Flag M2 and anti-HA 3F10, targeting respectively Flag-PEN2 and APH1aL-HA) were compared to exclude a possible heterogeneity of γ-secretase complexes potentially generated under such conditions. As shown in Figure 3B, the anti-Flag and anti-HA resins immunoprecipitated similar levels of NCT, PS1 and PEN2 and the activity of the γ-secretase complexes was similar regardless of the affinity resins used (Figures 3B). In agreement with the assays performed using microsomal extracts (Figure 3A), those performed with semi-purified complexes revealed that FAD-linked mutants drastically reduced AICD generation, when compared to PS1-WT (Figure 3B). Furthermore, levels of Aβ1–40 produced from PS1-L166P, PS1-ΔE9 and P436Q variants fell respectively to ∼6%, ∼1.5% and ∼0.5%, when compared to these produced from PS1-WT γ-secretase. Following the same tendency, Aβ1–42 levels generated from PS1-L166P, PS1-ΔE9 and PS1-P436Q γ-secretase variants were respectively estimated to ∼20%, ∼5% and ∼3% of these produced with PS1-WT-containing complexes. The Aβ1-42/Aβ1–40 ratio measured for WT γ-MEFs was consistent with our cell-based data (compare Figures 2D and 3C), validating our in vitro experimental conditions. Interestingly, an overall Aβ1–42/Aβ1–40 ratio of ∼1 was estimated for all PS1 mutants, with the exception of P436Q#4 (∼2). However, in the latter, the ratio may have been overestimated because the Aβ1–40 levels measured were at the detection limit. This similarity in the Aβ1–42/Aβ1–40 ratios generated by the different mutants has, to the best of our knowledge, never been observed in previous studies.

### Highly purified γ-secretase complexes with FAD-linked PS1 mutants display a loss-of-function phenotype

**Figure 4:**
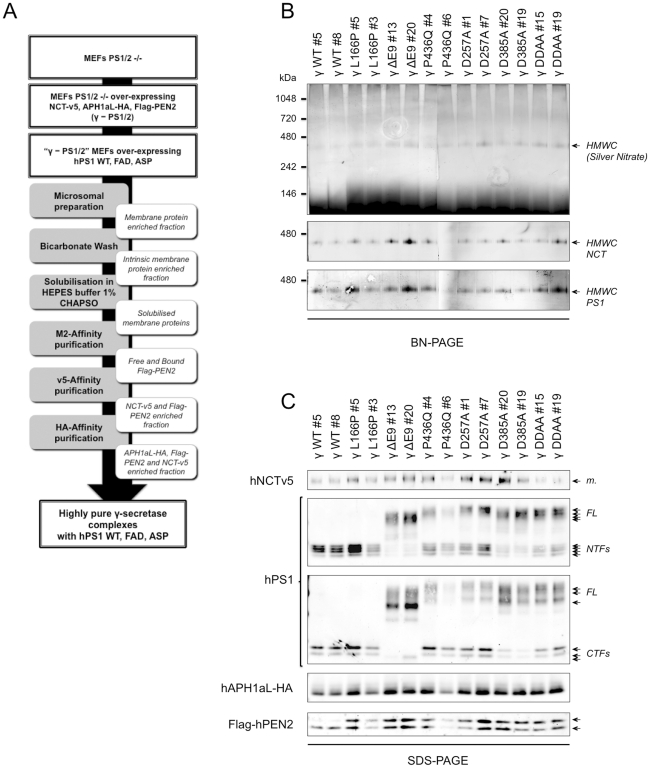
High-grade purification of human γ-secretase complexes with FAD-linked PS1 mutants. (A) Schematic representation of the γ-secretase purification process. Briefly, Presenilin double-knockout MEFs were used to first generate cell lines that stably overexpress human γ-secretase complexes containing different PS1 variants. Next, these cell lines were used for a multi-step purification procedure as described in the material and methods. (B) Blue-Native PAGE analysis of purified γ-secretase complexes made of different PS1 variants. Equal volumes of the different purified γ-secretase preparations were separated by native-PAGE on a 4–16% Bis-Tris gel, and stained with silver nitrate (top panel), or immunostained for NCT (NCT164, middle panel) or PS1-NTF (ab10281, bottom panel) as indicated. γ-Secretase complexes appeared on the gel as high molecular weight complexes (HMWCs) of ∼350 kDa. Note that the levels of HMWCs were similar for all clones. (C) Equal volumes of purified γ-secretase complexes with FAD-linked PS1 mutants were separated under denaturing conditions (SDS-PAGE) and immunostained with anti-NCT (NCT164), anti-PS1-NTF (MAB1563), anti-PS1-CTF (MAB5232), anti-HA (3F10), or anti-Flag (M2) antibodies. Two independent purifications were performed on each clone with similar results. A representative dataset is shown.

**Figure 5:**
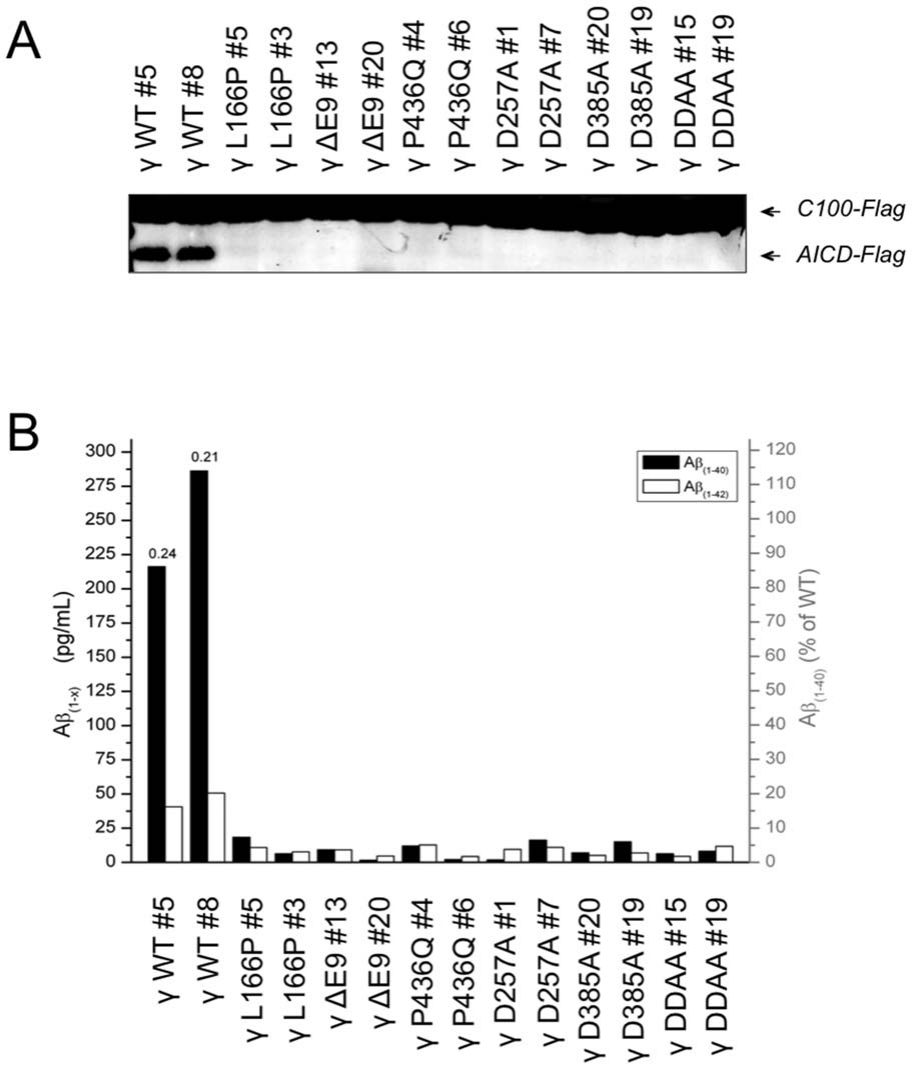
Enzymatic activity of highly purified γ-secretase complexes with FAD-linked or aspartate PS1 mutants. Equal amounts of the different purified γ-secretase preparations characterized in Figure 4 were tested for activity on C100-Flag, as described in Figure 3. The resulting cleavage products were separated by SDS-PAGE and detected by immunostaining with an anti-Flag antibody (M2) for C100-Flag or AICD-Flag (A), and by sandwich ELISA for Aβ1–40 or Aβ1–42 (B). Note that the levels of Aβ produced from FAD-linked γ-secretase complexes were all in the non-linear range of the ELISA standards, close to the detection limit. Whenever possible, Aβ1–42/Aβ1–40 ratios were quantified and indicated on the top of the bars. Two independent purifications were performed on each clone and similar results were obtained. A representative dataset is shown.

In order to further investigate how PS1 variants affect APP-CTF cleavage and to validate our observations performed in semi-purified conditions, we purified to homogeneity γ-secretase complexes from the selected clones described in Figure 1. The purification process (described in details under Materials & Methods and depicted in Figure 4A) involved three sequential affinity purifications starting from microsomal extracts. Since all cell lines were generated on a PS knockout background, this strategy allowed us to purify exclusively and selectively γ-secretase complexes made of human components. As highlighted in Figure 4B, our protocol led to the production of stable, high molecular weight γ-secretase complexes (HMWCs) with an apparent molecular mass on Blue Native (BN)-PAGE of ∼350 kDa, which is consistent with previous reports [26], [27], [37]. The silver stained particles on the BN gel confirmed the high purity of these complexes (Figure 4B, upper panel), for which the identity was established by cross-reactivity with antibodies specific to NCT and PS1 (Figure 4B, lower panels). As the main goal of this experiment was to compare the efficacy of the different γ-secretase complexes to process APP-based substrates, it is important to note that the final yields of the complexes purified from the different clones were very similar (Figure 4B), thus facilitating further comparative analyses. The immunoblot analysis of the purified complexes (Figure 4C) confirmed the presence of all core components (NCT-V5, APH1aL-HA, Flag-PEN2 and PS1) and globally recapitulates the maturation processes observed in the Figure 1. Interestingly, the relative PS1-NTF/CTF to PS1-FL ratios in the purified γ-secretase complexes containing the three PS1 dominant-negative or the PS1-P436Q variants were higher than those estimated in the whole cell extracts before purification (Figure 1A), indicating that endoproteolysis of PS1 was not completely abolished by these mutations. To assess how FAD-linked mutations in PS1 affect γ-secretase activity in these purified complexes, we performed *in vitro* assays by using C100-Flag substrate (Figure 3A) and equal amounts of the different purified complexes, immediately after the purification to avoid freezing/thawing cycles that might affect enzymatic activity. As shown in Figure 5A, γ-secretase activity based on AICD-Flag production was easily detected in complexes purified from the two PS1-WT clones. This is in sharp contrast to the other purified complexes that did not generate detectable AICD under our experimental conditions (Figure 5A). Next, sandwich ELISAs directed against Aβ1–40 and Aβ1–42 were performed in order to further characterize the specific activity of the purified complexes. Reflecting the levels of AICD and considering the detection limits of our sandwich ELISA, the production of both Aβ species by γ-secretase complexes containing PS1-WT was at least 10-fold higher than in complexes with the FAD-linked or dominant-negative PS1 variants (Figure 5B). The Aβ1–42/Aβ1–40 ratio measured in PS1-WT γ-secretase complexes (∼0.22) was similar to that previously reported [26], [27], thus excluding a possible qualitative alteration of γ-secretase activity with this protocol. Together, our results strongly support the hypothesis that the pathogenic L166P, ΔE9, and P436Q mutations in *PSEN1* cause a drastic loss (at least 90% as estimated by ELISA) of γ-secretase activity.

## Discussion

Several conflicting results as to how Alzheimer's disease-linked mutations in *PSEN1* affect the processing of APP by γ-secretase have been reported. First, it has been previously reported that transgenic animals with FAD-linked PS1 mutations show increasing brain levels of Aβ1–42 [15], [38]. Since Aβ1–42, the first Aβ specie deposited in the brain of AD patients [16], [39], is more prone to aggregation when compared to shorter Aβ species [40], [41], [42], it has been implicated in the seeding of amyloid plaques in AD patients with PSEN1 mutations [43]. This was further validated *in vivo* as the overexpression of PS1 mutants in APP transgenic mice accelerated the rate of Aβ accumulation and deposition in the brain [44], [45]. However, Bentahir and colleagues challenged this view by showing that several *PSEN1* mutations were also capable to decrease total Aβ production in PS1/PS2 knockout cells [23]. These findings suggested that endogenous PS1 and PS2 may influence the mutant phenotype in cells or *in vivo*. By extension, we hypothesized that the other components of γ-secretase, namely APH1, NCT and PEN2, may influence the mutant phenotype as well. Indeed, γ-secretase complexes are heterogeneous in composition due to the existence of two human APH1 genes, *APH1a* and *APH1b*, and two splicing isoforms of APH1a (S and L), as well as two presenilin genes, *PSEN1* and *PSEN2*. Therefore, it is plausible that a single mutation in *PSEN1* confers different catalytic properties to distinct γ-secretase subtypes. This hypothesis is supported by recent investigations showing that APH1 variants can modulate Aβ profiles. When compared to APH1aS or L, overexpression of APH1b in MEF knockout for all APH1 genes led to increased production of longer Aβ species [24]. With this regard, it is important to note that all four mouse variants of APH1 are expressed in the MEF cell line used in previous studies [23] and employed here (cf. Figure S3). Since the phenotype of PS1 mutations have mainly been assessed *in vivo* or in cell-based systems, we investigated the effects of FAD-linked PS1 mutants on the processing of APP-CTFs in cell-free systems, by using semi-purified and purified enzymatic complexes isolated from MEF PS1/PS2 double knockout cells stably overexpressing differentially tagged human γ-secretase components. Under these conditions, the biochemical and functional properties of γ-secretase complexes bearing either FAD-linked PS1 mutants (L166P, ΔE9, and P436Q), dominant-negative forms of PS1 or wild-type PS1 were characterized. The activity of γ-secretase with PS1-WT was similar to that reported for γ-secretase purified from our CHO cells overexpressing NCT-V5, APH1aL, Flag-PEN2 and PS1-WT [26], [27], that resulted in Aβ1–42/Aβ1–40 ratios between 0.1 and 0.3. In contrast, we found a major loss in the activity of γ-secretase complexes containing either the dominant-negative PS1 variants or the FAD-linked PS1 mutants. Although a total loss of activity was expected for the dominant negative forms of γ-secretase [5], [6], [32], the drastic loss of activity seen here with the FAD-linked PS1 mutants was unexpected. Indeed, the PS1-L166P variant has previously been reported to increase Aβ1–42 levels both *in vivo* [46] and *in vitro* [20], in the presence of endogenous PS1, PS2 and APH1 components. In contrast, Bentahir and colleagues found that PS1-L166P decreased both Aβ1–40 and Aβ1–42 production in a PS knockout background. These results are consistent with our *in vitro* data, although the reduction in Aβ1–42 and Aβ1–40 production was more pronounced in our system. However, they differ from our cell-based data in which we observed an increase in Aβ1–42 associated with a decrease in Aβ1–40. Taken together, these data suggest that the overexpression of the other human components of γ-secretase can influence the phenotype of FAD-linked mutations. Another possible explanation for these discrepancies comes from the use, in previously described cellular systems, of APP carrying the *Swedish* mutation (K670M/N671L) [20], [23], [46]. Initially, this APP variant was shown to enhance the production of all Aβ species by favouring its β-secretase cleavage [47], [48]. However, Munter and colleagues recently demonstrated that the APP *Swedish* mutation can also affect the specificity of the γ-secretase cleavage [49]. In particular, these authors showed that over-expression of APP *Swedish* in a neuronal cell line led to a 4-fold increase in secreted Aβ42, associated with only a 2-fold increase of total Aβ, compared with the wild-type APP. Therefore, one cannot exclude the possibility of differential interactions between PS1-WT or PS1 variants and different APP variants, as suggested earlier [50].

As far as the PS1-ΔE9 mutant is concerned, previous cell-based studies have reported a decrease in Aβ40 levels associated with an increase in Aβ42 levels [51], [52], or no changes in Aβ40 levels despite increased Aβ42 levels [53], or a decrease in both Aβ species [23]. In the present study, Aβ1–40, Aβ1–42, as well as AICD levels generated from purified γ-secretase complexes with PS1-ΔE9 were close to the limit of detection, resulting in a loss of at least 95% of the γ-secretase activity compared to wild-type PS1. Similarly, we found a near to complete loss of γ-secretase activity for complexes with the aggressive PS1-P436Q mutant (mean onset: 28.3 years) [54]. Consistent with a recent study [35], and in contrast to PS1-L166P, the maturation of the full-length PS1-P436Q protein into PS1-NTF and PS1-CTF was impaired. Interestingly, the same study also reported that P436Q variant showed a decreased γ-secretase activity and Aβ40 and Aβ42 secretion in PS1/PS2 double knockout cells (∼75% or ∼50% of the control for Aβ40 or Aβ42, respectively), but to a lesser extent than what we measured in this study (more than 97% for both Aβ species).

Overall, our data show that, under our experimental conditions, the FAD-linked PS1 mutants cause a drastic loss of activity (at least 90%) in the highly purified and homogeneous as well as semi-purified γ-secretase complexes. This loss-of-function is asymmetrical as Aβ1–42 levels were relatively less affected than Aβ1–40 levels. Interestingly, the ratio between the two Aβ species was similar for the variants tested here (∼1), suggesting a common modulatory mechanism. Our findings further support a model in which the subtype of γ-secretase containing APH1aL and FAD-linked PS1 mutants generates a higher Aβ42/Aβ40 ratio compared to PS1-WT, as previously observed *in vivo* [15], [38]. Taken together, the overall reduction in Aβ levels strongly suggest that other regulatory mechanisms or cellular components may exist and account for the strong amyloid pathology observed in AD patients carrying these FAD-linked *PSEN1* mutations. In agreement with this hypothesis, we report that the phenotype of FAD-linked *PSEN1* mutations is different whether we consider the cell-based or the cell-free conditions. Indeed, even if Aβ42/Aβ40 ratios were globally increased in both experimental conditions, Aβ production was strikingly different.

One possible explanation would be that specific subtypes of γ-secretase complexes are less affected than others by *PSEN1* mutations. For instance, it remains unknown whether γ-secretase complexes carrying APH1b or APH1aS are similarly affected by *PSEN1* mutations. In support to that regard, Winkler and colleagues recently purified human γ-secretase with PS1-L166P and observed that such complexes, containing both APH1a and AHP1b, led to increased Aβ1–42 production associated with decreased Aβ1–40 production, as compared to the PS1-WT complexes. Another possible explanation would be that purified γ-secretase complexes lack binding partners modulating the processing of APP-based substrates, due to purification conditions affecting physical interactions of proteins. These include TMP21 or the recently reported γ-secretase activating protein (GSAP) [12], [13]. Further biochemical analyses are needed to test this hypothesis.

The loss of activity in γ-secretase containing PS1 variants also leads to decreased generation of AICD. As AICD is involved in the transcriptional regulation of several genes, including the neprilysin [55], and the lipoprotein receptor, LRP1 [11], it is likely that mutations in *PSEN1* impair the regulation of these genes *in vivo*. For instance, it is possible that unknown genes transcriptionally regulated *in vivo* by the AICD might influence Aβ metabolism in return. In support to these views, Veeraraghavalu and colleagues recently demonstrated that Notch signalling was impaired in transgenic mice overexpressing PS1 mutants, albeit in the presence of endogenous PS1-WT and all APH1 isoforms [56]. They found decreased self-renewal and differentiation of neuronal precursor cells in the subventricular zone, suggesting that the loss-of-function phenotype of *PSEN1* mutations can also be observed in heterogeneous conditions. These results are reminiscent of the previous report from Saura and colleagues showing that conditional inactivation of both presenilins *in vivo* induces age-dependent neurodegeneration associated with memory impairment [57]. Altogether, the above-described data suggest that alternative pathways of neurodegeneration related to loss of γ-secretase functions are possible and relevant to AD. They further support the concept that modulating rather than inhibiting γ-secretase activity would be a more appropriate therapeutic strategy for AD [58]. Supporting this view, the γ-secretase inhibitor Semagacestat tested in phase III clinical trials not only failed to slow cognitive decline in patients with mild-to-moderate AD, but actually made it worse [59].

Collectively, our findings support a model in which FAD-linked mutations in *PSEN1* likely induce Aβ pathology by perturbing the relative ratio between Aβ species and by impairing developmental and cellular signalling pathways controlled by γ-secretase substrates. This deleterious dual effect might explain why FAD-linked *PSEN1* mutations cause early onset Alzheimer's disease. Since it has been established that Aβ and AICD are generated following the processing by γ-secretase at two distinct gamma- and epsilon-cleavage sites in APP [60], further investigation is now required to better understand whether and how FAD mutations in PS1 differentially affect the epsilon versus gamma-cleavage sites in APP, as well as in other known γ-secretase substrates.

## Materials and Methods

### DNA Constructs and mutagenesis

NCT-V5, Flag-PEN2, APH1aL-HA, wild-type PS1 human cDNAs (PS1-WT) were obtained from D. Selkoe (Harvard Medical School and Brigham and Women's Hospital, Boston, MA, USA). PS1 ΔE9 cDNA was obtained from C. Saura (Universitat Autònoma de Barcelona, Spain). PS1 D257A and PS1 D385A cDNAs were obtained from M. Wolfe (Harvard Medical School and Brigham and Women's Hospital, Boston, MA, USA). Mutations in *PSEN1*, namely, L166P and P436Q, were generated by PCR-based mutagenesis on the plasmid pcDNA3.1/Zeo(+)-PS1-WT using T7 or BGH primers (specific to the plasmid DNA sequence) together with the corresponding primers: *L166P Rv*: 5′ CAG CAA CAA Taa gct tGA TAT AAT AGG 3′; *L166P Fw*: 5′ CTT ATT ATA TCa agc ttA TTG TTG CTG 3′; *P436Q Rv*: 5′ TCG AGT TTA Gaa gct tTC TTG AAA ATG GCA AGG AG 3′; *P436Q Fw*: 5′ TCG AGT TTA Gaa gct tTG CCA GCT CTT CAA ATC TCC 3′. The sequences corresponding to enzyme restriction sites are given in small letters. The PCR fragments were next digested with *Bam*HI/*Hin*dIII and *Hin*dIII/*Xho*I respectively, and were subcloned into pcDNA3. PS1 D257A/D385A (DDAA) was obtained by subcloning the *Dra*I/*Xho*I digested fragment of PS1 D385A into pcDNA3.1-PS1 D257A. The APP based substrate SPA4CT-Flag, corresponding to the signal peptide of APP fused to the APP-C99-Flag with a small linker region (DA) [36], was obtained by removing the *Hin*dIII/*Eco*RI fragment of pcDNA3-APP695-Flag and inserting the following annealed primers: 5′ AGC TTA TGC TGC CCG GTT TG GCA CTG CTC CTG CTG GCC GCC TGG ACG GCT CGG GCG GAT GCA GAT GCA G 3′ and 5′ AAT TCT GCA TCT GCA TCC GCC CGA GCC GTC CAG GCG GCC AGC AGG AGC AGT GCC AAA CCG GGC AGC ATA 3′. All constructs were next subcloned into the self-inactivated vector pSIN-PGK-WHV cassette (a kind gift from R. Zufferey, Brain Mind Institute, EPFL, Lausanne, Switzerland). Inserts for plasmids pET21-C100-Flag, and pET21-C100-His were generated by PCR as previously described [27]. Plasmids pMD2G and psPAX2 were obtained from D. Trono (Global Health Institute, EPFL, Lausanne, Switzerland).

### Lentivector production

Replication-defective lentiviral particles were produced by a three-plasmid transient transfection of cells from the human embryonic kidney HEK 293T cell line [61]. Briefly, cells were incubated in 10 cm dishes until they reached 70% confluence and were transiently co-transfected by the calcium phosphate method, with 5 µg of envelope plasmid (pMD2G), 15 µg of packaging plasmid (psPAX2), and 20 µg of vector plasmids (pSIN-PGK-WHV). Cells were incubated overnight with DNA-calcium precipitates, washed twice with Dulbecco's modified Eagle's medium (DMEM, Invitrogen) and incubated with 7 mL of DMEM. After 24 h, the conditioned medium was harvested and cells were incubated a second time with 7 mL of DMEM for 24 h. Media were then pooled, passed through 0.45 µm filter, and stored at −80°C as 2 mL aliquots until use. A p24 ELISA (Zeptometrix corporation) was performed on each batch of media in order to evaluate the number of viral particles generated.

### Generation of stable cell lines through multiple gene transductions

Mouse Embryonic Fibroblasts (MEFs, obtained from B. De Strooper, Flanders University Institute of Biotechnology, Belgium) defective in *PSEN1* and *PSEN2* [30], [31] were cultivated in 10 cm dishes in DMEM, supplemented with 10% foetal bovine serum (FBS) and penicillin/streptomycin (P/S) (Invitrogen). A first set of stable cell lines was generated by co-transducing NCT-V5, APH1aL-HA, and Flag-PEN2 containing lentiviral vectors (LV) repeatedly for two weeks at each passage (1/20). Ten clones were isolated by the limiting dilution method and were further screened for NCT-V5, Flag-PEN2 and APH1aL-HA expression by immunodetection. The clone that showed the highest expression of the three proteins (designated γ - PS1/2) was used as the parental cell line to generate the stable cell lines expressing different PS1 variants, by the same method and by using lentiviral vectors carrying human PS1-WT, PS1-L166P, PS1-ΔE9, PS1-P436Q, PS1-D257A, PS1-D385A, and PS1-DDAA. For each PS1 variant, five clones were selected for a full characterization and two of them were further used for γ-secretase purification. For SPA4CT overexpression experiments, 500,000 cells of each clone were exposed to the same arbitrary dose of lentiviral vectors carrying human SPA4CT-Flag in 2 mL of DMEM, 1% FBS, P/S and were plated in 6-wells plates for 72 h. The medium was next replaced by fresh DMEM, 2% FBS, P/S for 24 h. Finally, cell culture medium was harvested, supplemented with protease inhibitor cocktail (Roche), centrifuged for 3 min at 1,000× rpm and frozen at −80°C until further processing. Cells were washed in PBS and submitted to protein extraction.

### Protein extraction

Total protein extracts were prepared in 1% NP40 - HEPES buffer (50 mM HEPES, pH 7.0, 150 mM NaCl, 5 mM MgCl_2_, 5 mM CaCl_2_), supplemented with protease inhibitor cocktail (Roche), and were clarified by centrifugation for 1 h at 16,000× *g*, 4°C. Cell membranes were obtained as described below and solubilised in 1% CHAPSO - HEPES buffer and centrifuged at 16,000× *g* for 1 h. Supernatants corresponding to the microsomal protein extracts were harvested and stored at −80°C. Protein content of the extracts was estimated by the BCA protein assay reagent kit (Pierce).

### Multi-step purification of human γ-secretase complexes with FAD-linked PS1 variants

*1) Cell membrane preparation*. MEF PS1/PS2 DKO cells (1.0×10^9^ cells) expressing NCT-V5, Flag-PEN2, APH1aL-HA and different forms of PS1: wild type (WT), or with FAD-linked variants or lacking the critical aspartate residues, were collected from 15 cm dishes and fully resuspended in 40 mL of MES buffer (50 mM MES, pH 6.0, 150 mM NaCl, 5 mM MgCl_2_, 5 mM CaCl_2_), supplemented with protease inhibitor cocktail (Roche). Next, cells were passed four times through a high-pressure homogenizer (Emulsiflex-C5, Avestin Inc, ON Canada) at a pressure greater than 1,000 psi. Nuclei and unbroken cells were removed by centrifugation at 3,000× *g* for 20 min at 4°C in a Beckman Coulter Allegra X-15R centrifuge. The supernatant was collected and centrifuged at 100,000× *g* for 1 h at 4°C in a SW32Ti rotor using a Beckman Coulter Optima L-80 ultracentrifuge to recover the membrane preparation in the pellet. *2) Bicarbonate wash*. The membrane pellet was fully resuspended in 1.6 mL of ice cold sodium bicarbonate buffer (0.1 M NaHCO_3_, pH 11.3) by pipetting up and down at least 30 times, and incubated at 4°C for 20 min. The washed membranes were then centrifuged at 100,000× *g* for 1 h at 4°C and stored at −80°C until use. *3) Solubilisation of γ-secretase complexes*. The bicarbonate-washed membranes were fully resuspended in 1.7 mL of 1% CHAPSO - HEPES buffer by pipetting up and down at least 30 times. The membranes were then incubated at 4°C for 1 h. To pellet the insoluble material, the solution was centrifuged at 16,000× *g* at 4°C for 1 h, the pellet was discarded, and the supernatant saved. This lysate is defined as “solubilised γ-secretase preparation”. Next, these freshly prepared solubilised preparations were used for affinity purification of γ-secretase complexes as described below. *4) Anti-Flag M2 affinity purification*. The solubilised γ-secretase preparations were first diluted 1∶2 with HEPES buffer, and further diluted 1∶6 with 0.1% digitonin - TBS buffer (50 mM Tris-HCl, pH 7.4, 150 mM NaCl) (final detergent concentration: 0.08% CHAPSO, 0.08% digitonin) and incubated overnight at 4°C with agitation after adding 200 µL of anti-Flag M2 affinity resin beads (Sigma-Aldrich) that had been pre-equilibrated in 0.1% Digitonin - TBS buffer. The beads were washed 3 times in the same buffer, and the bound proteins were eluted over 4 h at 4°C with 0.4 mL of this buffer containing 200 µg/mL of Flag peptides (Sigma-Aldrich). This elution step was repeated once for 1 h at 4°C and the eluted fractions were pooled and designated “M2 pooled 800 µL fractions”. *5) Anti-V5 affinity purification*. The M2 pooled fractions (volume made up to 1 mL with 0.1% Digitonin - TBS buffer) were next incubated overnight at 4°C under agitation with 200 µL of anti-V5 affinity resin beads (Sigma-Aldrich), pre-equilibrated in 0.1% digitonin-TBS buffer. The beads were washed 3 times in the same buffer, and the bound proteins were eluted for 1 h at 4°C with 0.4 mL of this buffer containing 500 µg/mL of V5 peptides (Sigma-Aldrich). This elution step was repeated 4 times, and the eluted fractions were pooled and designated “V5 pooled 1.6 mL fractions”. *6) Anti-HA affinity purification*. As a final purification step, the V5 pooled fractions (volume made up to 1.8 mL with 0.1% digitonin - TBS buffer) were next incubated overnight at 4°C under agitation with 200 µL of anti-HA affinity resin beads (Sigma-Aldrich), pre-equilibrated in 0.1% digitonin - TBS buffer. The beads were washed 3 times in the same buffer, and the bound proteins were eluted overnight at 4°C with 0.2 mL of this buffer containing 200 µg/mL of HA peptides (Sigma-Aldrich). This elution step was repeated once, for 1 h at 4°C, and the two eluted fractions were kept separately, designated “HA fraction E1” and “HA fraction E2”.

### Co-immunoprecipitation

Co-IPs were performed on microsomal protein extracts. Briefly, 100 µg of proteins were diluted in a final volume of 1 mL of 1% CHAPSO - HEPES extraction buffer containing protease inhibitors (Roche), and 50 µL of pre-equilibrated anti-Flag or anti-HA affinity beads (Sigma-Aldrich) were added. Samples were next incubated overnight at 4°C on a rotator wheel and were washed three times in 0.2% CHAPSO - HEPES buffer. Finally, beads were resuspended in 50 µL of 0.2% CHAPSO - HEPES buffer and were used for *in vitro* γ-secretase assays.

### In vitro γ-secretase activity assays

*In vitro* γ-secretase activity assays were performed as previously described [27]. γ-Secretase APP-based substrates were expressed in *E. coli* transfected with plasmids pET21-C100-Flag or pET21-C100-His as a fusion protein consisting of a Met for translation initiation, and either the Flag tag sequence (C100-Flag) or the His tag sequence (C100-His), and were affinity purified using an anti-Flag resin (M2, Sigma-Aldrich) or an Ni-NTA agarose resin (Invitrogen), respectively. *In vitro* assays were performed at 37°C for 4 h, with 1 µM of recombinant substrate, 0.025% phosphatidylethanolamine (PE) and 0.1% phosphatidylcholine (PC). γ-Secretase activity was quantified by measuring the amount of AICD and Aβ generated during the reaction, by immunoblot or sandwich ELISA respectively, as described below.

### SDS-PAGE, Native PAGE, Western blotting and antibodies

Total or microsomal protein extracts were resolved by electrophoresis on NuPAGE® Novex® 4–12% Bis-Tris Gels (Invitrogen) or on standard 12% acrylamide/bisacrylamide Tris Glycine gels for SDS-PAGE analysis. Purified γ-secretase was resolved by electrophoresis on NativePAGE™ Novex® Bis-Tris 4–16% Gels for Blue Native (BN)-PAGE analysis (Invitrogen). Silver staining was performed directly on gel according to manufacturer instructions (Biorad). For immunoblot analysis, gels were transferred to nitrocellulose or PVDF membranes (Whatman), and probed with the following antibodies: anti-Nicastrin NCT164 (BD Bioscience), anti-V5-tag for NCT-V5 (Covance), MAB1563 (Millipore) or ab10281 (Abcam) for PS1 NTF, MAB5232 for PS1 CTF (Millipore), 3F10 for APH1aL-HA (Roche), anti-Flag M2 for Flag-PEN-2 or C100-Flag (Sigma-Aldrich), A8717 for APP CTF (Sigma-Aldrich), and A2066 for β-actin (Sigma-Aldrich). Anti-mouse/rabbit/rat IgG conjugated to Alexa 680 were purchased from Invitrogen. The Odyssey infrared imaging system (LICOR) was used to detect the fluorescent signal.

### Quantification of Aβ peptides

Aβ peptides from the γ-secretase assays described above were quantified by sandwich ELISA according to the protocol provided by the manufacturer. Three kits were used to detect human Aβ1–40 (Invitrogen KHB3481) and Aβ1–42 (Invitrogen KHB3544 and Wako 269-64401).

## Supporting Information

Figure S1 **Characterization of stable cell lines overexpressing human FAD-linked PS1 variants in MEF PS1/2^−/−^.** Presenilin double-knockout mouse embryonic fibroblasts were stably transduced with lentiviral vectors carrying genes encoding hPS1 variants harbouring FAD-linked mutations or mutations in the catalytic aspartate residue(s), or PS1-WT, and cloned. Two clones per PS1 variant were selected for characterization. (A) Whole cell protein extracts of the different cell lines were prepared in 1% NP40-HEPES buffer, separated by SDS-PAGE on 12% Tris-Glycine gels and analysed by immunostaining with various antibodies to detect the endogenous γ-secretase core components: NCT (NCT164), hPS1 (NTF, MAB1563), and PEN2 (UD1), and with an antibody for APP (A8717). β-Actin was used as a loading control. Each lane represents one selected clone. (B) γ-Secretase activity assays were performed with microsomal extracts prepared in 1% CHAPSO-HEPES buffer. Equal protein levels from the different extracts were diluted to 0.25% CHAPSO-HEPES buffer and incubated for 4 h at 37°C with lipids and 1 µM of recombinant human APP-based substrate (C100-Flag). Samples were analyzed by SDS-PAGE and immunostained with anti-Flag (M2) or anti-PS1 (MAB1563). PS1 immunostaining was used to assess the amount of input material. * Indicates a non-specific band, which was not detected in microsomal protein extracts of the same cell lines using the same antibody (MAB1563) (B). CTF: C-terminal fragment, FL: full-length, im.: immature NCT; m.: mature NCT, N: N-glycosylated, NTF: N-terminal fragment, O: O-glycosylated.
(TIF)

Figure S2 **APP-CTF profiles in WT MEF, MEF PS1/2^−/−^ and γ - PS1/2 transduced with the SPA4CT construct.** WT MEF, MEF PS1/2^−/−^ and γ - PS1/2 were transduced with an APP-based substrate corresponding to the 99 C-terminal residues of human APP fused to the APP signal peptide in N-terminus (SPA4CT [36]). Cell proteins were extracted in 1% NP40-HEPES buffer, separated by SDS-PAGE on 12% Tris-Glycine gels and analysed by immunostaining with an antibody targeting the C-terminal part of APP (A8717).
(TIF)

Figure S3 **APH1 isoforms expressed in MEF PS1/2^−/−^.** Total RNAs were extracted from presenilin double-knockout MEFs, using standard procedures (Qiagen RNAeasy kit) and were quantified by spectrophotometry. One microgram of total RNAs was reverse transcribed for 1 h at 42°C by using the ImProm-II reverse transcription system (Promega) and oligo-dT primer in a final volume of 20 µL. PCR was next performed for each APH1 isoform on 1 µL of RT reaction by using the Roche PCR kit under standard conditions and the primers described in Table S1. The following cycling conditions were applied for all reactions: 94°C, 3 min; 30 cycles of [94°C, 30 s; 58°C, 30 s; 70°C, 40 s]; 70°C, 10 min. PCR products were separated on a 2% agarose gel and visualized using Alpha Innotech UV imager.
(TIF)

Table S1 **Primers used to detect APH1 isoforms in MEF PS1/2^−/−^.** F: Forward, R: Reverse.
(DOC)
