# Supplementary figures and images for: Alzheimer's Disease-Linked Mutations in Presenilin-1 Result in a Drastic Loss of Activity in Purified γ-Secretase Complexes
Source: PLoS One. 2012 Apr 18;7(4):e35133. doi: 10.1371/journal.pone.0035133 (PMC3329438; doi:10.1371/journal.pone.0035133)

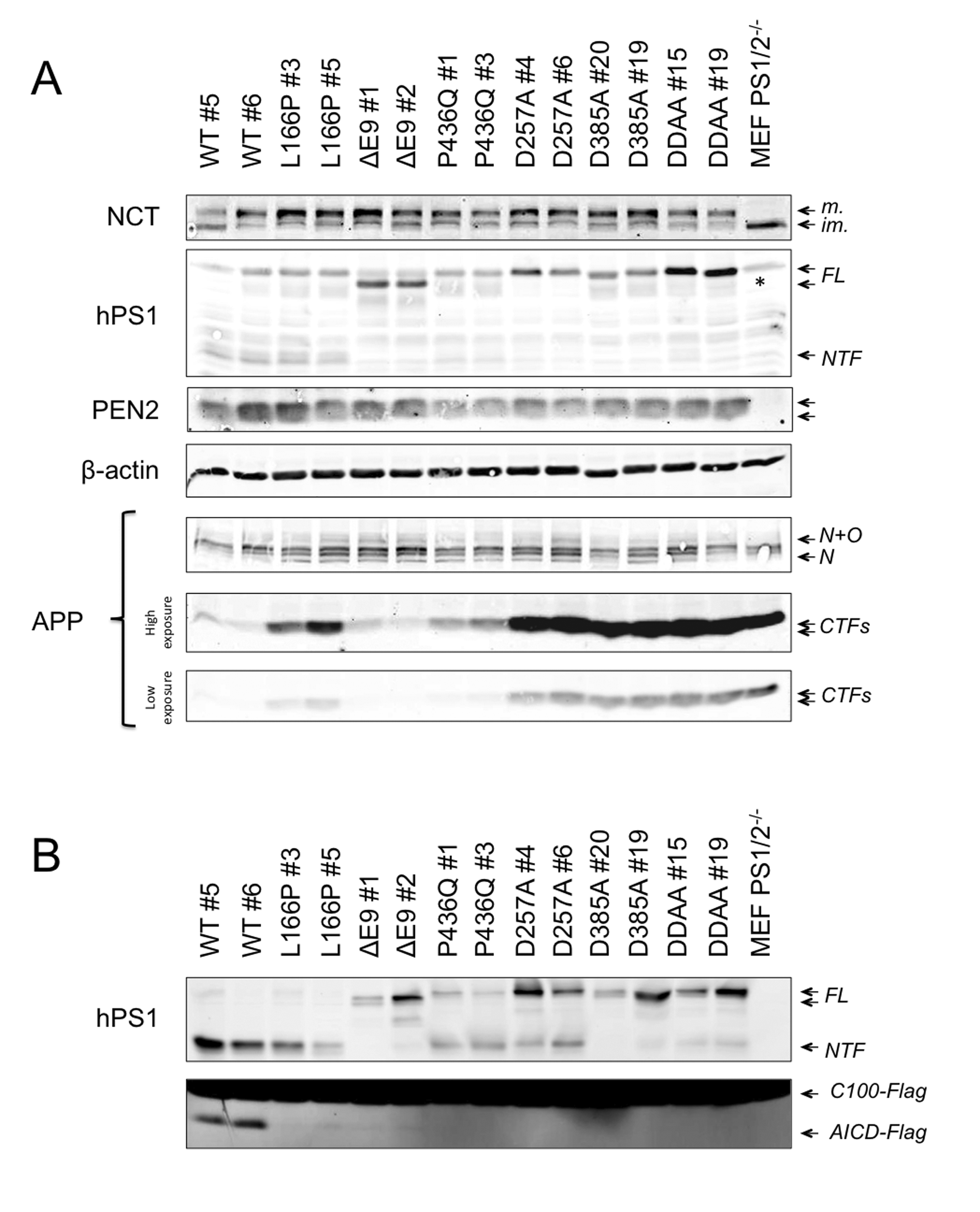

Supplement: Figure S1 — Characterization of stable cell lines overexpressing human FAD-linked PS1 variants in MEF PS1/2−/−. Presenilin double-knockout mouse embryonic fibroblasts were stably transduced with lentiviral vectors carrying genes encoding hPS1 variants harbouring FAD-linked mutations or mutations in the catalytic aspartate residue(s), or PS1-WT, and cloned. Two clones per PS1 variant were selected for characterization. (A) Whole cell protein extracts of the different cell lines were prepared in 1% NP40-HEPES buffer, separated by SDS-PAGE on 12% Tris-Glycine gels and analysed by immunostaining with various antibodies to detect the endogenous γ-secretase core components: NCT (NCT164), hPS1 (NTF, MAB1563), and PEN2 (UD1), and with an antibody for APP (A8717). β-Actin was used as a loading control. Each lane represents one selected clone. (B) γ-Secretase activity assays were performed with microsomal extracts prepared in 1% CHAPSO-HEPES buffer. Equal protein levels from the different extracts were diluted to 0.25% CHAPSO-HEPES buffer and incubated for 4 h at 37°C with lipids and 1 µM of recombinant human APP-based substrate (C100-Flag). Samples were analyzed by SDS-PAGE and immunostained with anti-Flag (M2) or anti-PS1 (MAB1563). PS1 immunostaining was used to assess the amount of input material. * Indicates a non-specific band, which was not detected in microsomal protein extracts of the same cell lines using the same antibody (MAB1563) (B). CTF: C-terminal fragment, FL: full-length, im.: immature NCT; m.: mature NCT, N: N-glycosylated, NTF: N-terminal fragment, O: O-glycosylated. (TIF) [file pone.0035133.s001.tif]

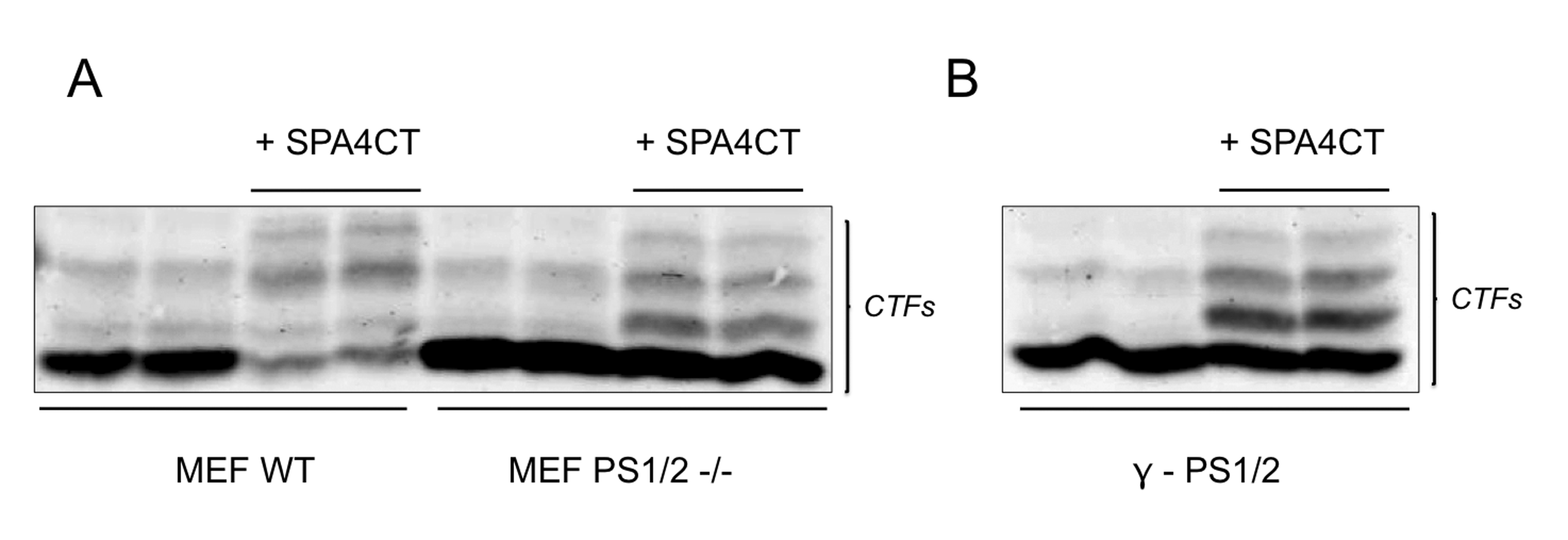

Supplement: Figure S2 — APP-CTF profiles in WT MEF, MEF PS1/2−/− and γ - PS1/2 transduced with the SPA4CT construct. WT MEF, MEF PS1/2−/− and γ - PS1/2 were transduced with an APP-based substrate corresponding to the 99 C-terminal residues of human APP fused to the APP signal peptide in N-terminus (SPA4CT [36]). Cell proteins were extracted in 1% NP40-HEPES buffer, separated by SDS-PAGE on 12% Tris-Glycine gels and analysed by immunostaining with an antibody targeting the C-terminal part of APP (A8717). (TIF) [file pone.0035133.s002.tif]

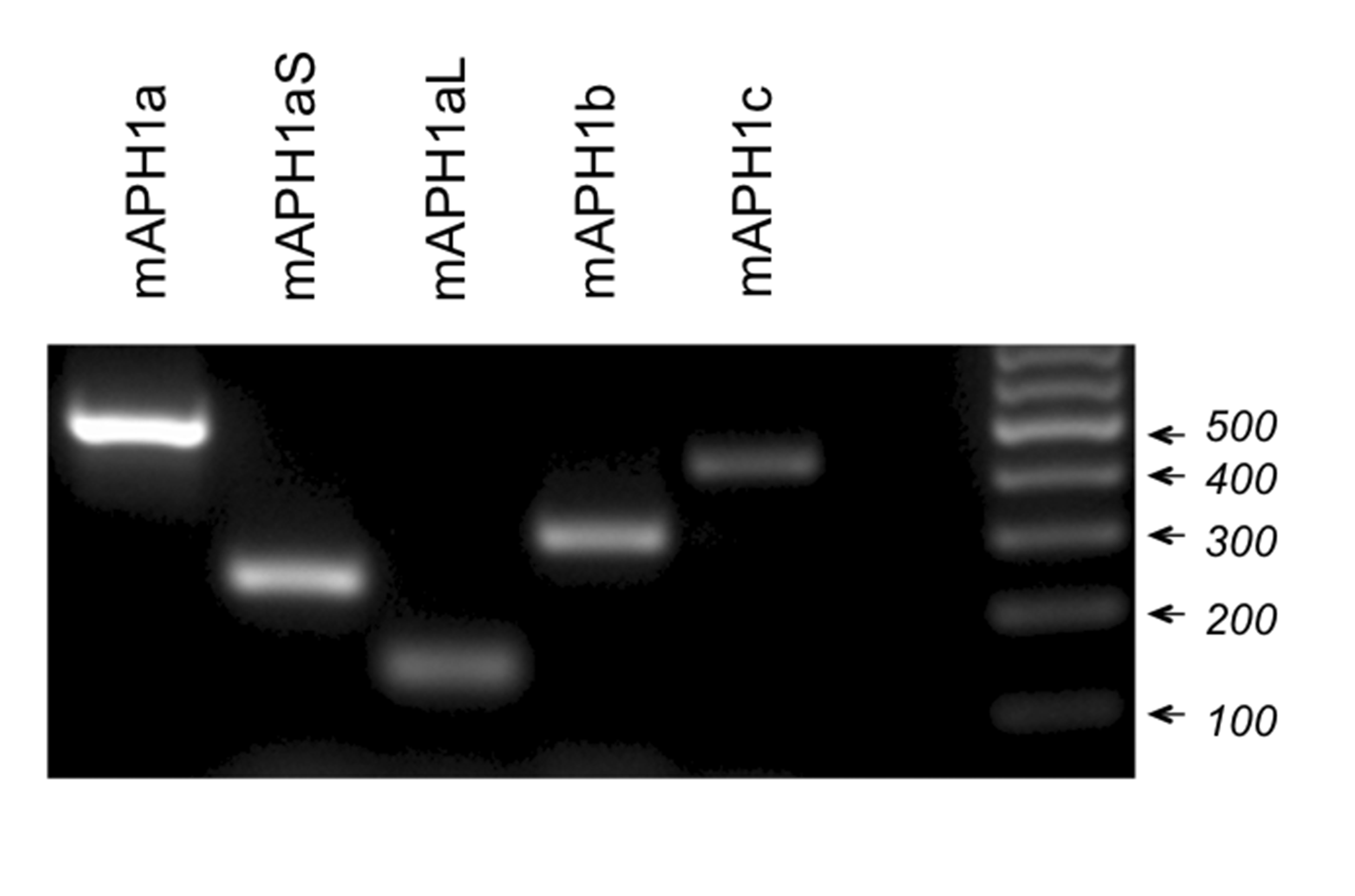

Supplement: Figure S3 — APH1 isoforms expressed in MEF PS1/2−/−. Total RNAs were extracted from presenilin double-knockout MEFs, using standard procedures (Qiagen RNAeasy kit) and were quantified by spectrophotometry. One microgram of total RNAs was reverse transcribed for 1 h at 42°C by using the ImProm-II reverse transcription system (Promega) and oligo-dT primer in a final volume of 20 µL. PCR was next performed for each APH1 isoform on 1 µL of RT reaction by using the Roche PCR kit under standard conditions and the primers described in Table S1. The following cycling conditions were applied for all reactions: 94°C, 3 min; 30 cycles of [94°C, 30 s; 58°C, 30 s; 70°C, 40 s]; 70°C, 10 min. PCR products were separated on a 2% agarose gel and visualized using Alpha Innotech UV imager. (TIF) [file pone.0035133.s003.tif]
