# Supplementary material for: Alzheimer's Disease-Linked Mutations in Presenilin-1 Result in a Drastic Loss of Activity in Purified γ-Secretase Complexes
Source: PLoS One. 2012 Apr 18;7(4):e35133. doi: 10.1371/journal.pone.0035133 (PMC3329438; doi:10.1371/journal.pone.0035133)
Supplement: Table S1 — Primers used to detect APH1 isoforms in MEF PS1/2−/−. F: Forward, R: Reverse. (DOC) [file pone.0035133.s004.doc]

**Table S1: Primers used to detect APH1 isoforms in MEF PS1/2^-/-^**

| **Gene** | **Amplicon size (bp)** | **Primer sequence** | **Direction*** |
| --- | --- | --- | --- |
| mAPH1a (long and short) | 478 | 5' GGTCTGGTTCATCTTGGTCC 3' | F |
|  |  | 5' ATACCAGGGGTTCAGGAATGTC 3' | R |
| mAPH1a short | 236 | 5' ATTCCTGAACCCCTGGTATG 3' | F |
|  |  | 5' GCGAGGAGACGGAGGATGAG 3' | R |
| mAPH1a long | 139 | 5' ATTCCTGAACCCCTGGTATG 3' | F |
|  |  | 5' GTCCTCCTGCCGGCGGCAC 3' | R |
| mAPH1b | 299 | 5' GCCCGCGCTCGCTCTTTATG 3' | F |
|  |  | 5' GTCGCATCGAGGGTGCTGTC 3' | R |
| mAPH1c | 415 | 5' CGCTAAGAAATCGTCCCAGTC 3' | F |
|  |  | 5' CAGTCGCATCGAGGGTGCTATG 3' | R |

* F: Forward, R: Reverse
